# When enough is enough: Optimising monitoring effort for large‐scale wolf population size estimation in the Italian Alps

**DOI:** 10.1002/ece3.70204

**Published:** 2024-08-21

**Authors:** M. V. Boiani, P. Dupont, R. Bischof, C. Milleret, O. Friard, M. Geary, E. Avanzinelli, A. von Hardenberg, F. Marucco

**Affiliations:** ^1^ Department of Biological Sciences Conservation Biology Research Group, University of Chester Chester UK; ^2^ Faculty of Environmental Sciences and Natural Resource Management Norwegian University of Life Sciences Ås Norway; ^3^ Department of Life Sciences and Systems Biology University of Turin Turin Italy; ^4^ Centro Grandi Carnivori, Ente di Gestione Aree Protette Alpi Marittime Valdieri Cuneo Italy; ^5^ Department of Earth and Environmental Sciences University of Pavia Pavia Pavia Italy

**Keywords:** adaptive management, large carnivore, large scale monitoring, long‐term monitoring, monitoring optimisation, non‐invasive sampling, spatial capture‐recapture, wolf population

## Abstract

The ongoing expansion of wolf (*Canis lupus*) populations in Europe has led to a growing demand for up‐to‐date abundance estimates. Non‐invasive genetic sampling (NGS) is now widely used to monitor wolves, as it allows individual identification and abundance estimation without physically capturing individuals. However, NGS is resource‐intensive, partly due to the elusive behaviour and wide distribution of wolves, as well as the cost of DNA analyses. Optimisation of sampling strategies is therefore a requirement for the long‐term sustainability of wolf monitoring programs. Using data from the 2020–2021 Italian Alpine wolf monitoring, we investigate how (i) reducing the number of samples genotyped, (ii) reducing the number of transects, and (iii) reducing the number of repetitions of each search transect impacted spatial capture‐recapture population size estimates. Our study revealed that a 25% reduction in the number of transects or, alternatively, a 50% reduction in the maximum number of repetitions yielded abundance estimates comparable to those obtained using the entire dataset. These modifications would result in a 2046 km reduction in total transect length and 19,628 km reduction in total distance searched. Further reducing the number of transects resulted in up to 15% lower and up to 17% less precise abundance estimates. Reducing only the number of genotyped samples led to higher (5%) and less precise (20%) abundance estimates. Randomly subsampling genotyped samples reduced the number of detections per individual, whereas subsampling search transects resulted in a less pronounced decrease in both the total number of detections and individuals detected. Our work shows how it is possible to optimise wolf monitoring by reducing search effort while maintaining the quality of abundance estimates, by adopting a modelling framework that uses a first survey dataset. We further provide general guidelines on how to optimise sampling effort when using spatial capture‐recapture in large‐scale monitoring programmes.

## Graphical abstract

The ongoing wolf recolonization is followed by the growing demand from administrations for increasingly up‐to‐date and accurate population estimates. Although the methods for obtaining such estimates are well known, there is now a growing interest in applying it over large spatial extents. However, the difficulty of repeating sampling surveys over time is a limitation that often makes it impossible to complete long‐term monitoring programmes. In this study, we used data from Alpine‐scale wolf monitoring conducted in 2020/2021 in northern Italy and subsampled the original dataset to assess the robustness of SCR estimates under different effort scenarios or reduced samples. Our results demonstrate the possibility of reducing monitoring effort even at large scales and for complex and elusive species such as wolves. They encourage the continuation of such studies by adapting the sampling design over the years and optimising it for a long‐term monitoring programme.

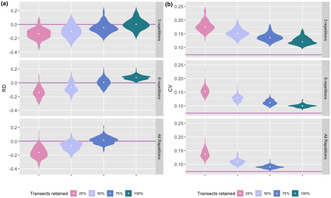

## 1 INTRODUCTION

Accurate estimates of demographic parameters such as population size and density are critical for wildlife management and conservation (Holland et al., 2012; Lindenmayer et al., 2022). Inaccurate population size estimates can lead to false interpretations of the impact of conservation or management interventions (e.g., culling or hunting). In addition to robust estimation methods, scientists have emphasised the need for long‐term monitoring programs to assess population trends over time and not just point estimates (Habel et al., 2014; White, 2019). However, reliable and effective long‐term monitoring of wildlife species is inherently complex and costly. This is especially true for expanding or recovering species, given that the area and number of individuals to keep track of increases over time (Marucco et al., 2023; Milleret et al., 2020).

The recent recolonization of Europe by the wolf (*Canis lupus*) (Boitani et al., 2022; Chapron et al., 2014) has led to a pressing demand from stakeholders and managers to quantify their abundance and distribution. These estimates are also required to meet legal obligations in Europe (Annex XVII of the Habitat Directive, 92/43/2000 CE) and are necessary given the conflictual relationship between human activities and predators (Kuijper et al., 2019; López‐Bao et al., 2015). This recurrent demand for population size estimates has sparked methodological advances in both data collection (Bohmann et al., 2014; Hodgson et al., 2018; Stephenson, 2020) and statistical analysis (Bischof et al., 2020; Blanc et al., 2014; Jiménez et al., 2016). Non‐invasive genetic sampling, which enables identifying individuals from DNA extracted from samples such as scats and hairs, has been successfully used to estimate wolf population sizes in different places (Bischof et al., 2020; López‐Bao et al., 2018; Marucco et al., 2009; Stenglein et al., 2010). Spatial Capture‐Recapture (SCR) (Borchers & Efford, 2008; Royle & Young, 2008) has in turn proved particularly suitable to analyse such data (Kéry, 2011). SCR models exploit the spatial distribution of non‐invasive genetic samples (NGS) to estimate individual activity centres and produce spatially explicit estimates of density for entire populations (Bischof et al., 2020). Nevertheless, SCR models are data‐hungry models, which require large datasets and a high sampling effort to obtain an adequate spatial representation (Dupont et al., 2019). There is an inherent trade‐off between sampling effort and the precision of population size estimates: higher effort leads to larger sample size, which in turn reduces uncertainty in estimates (Paterson et al., 2019). For widespread and large populations, this means collecting large numbers of NGS over large areas and thus high field effort and laboratory costs (e.g., genetic analysis). This level of monitoring effort is hardly sustainable in the long‐term.

In the Italian Alps, the wolf population was intensively sampled during the winter of 2020–2021. The sampling scheme consisted of numerous search transects systematically distributed over the entire Italian Alpine arch, which were repeatedly searched over the course of 6 months. This extensive search effort – 40,725 km walked by field staff and volunteers – led to the first landscape‐scale population density map and abundance estimate using SCR (Marucco et al., 2023). This work was made possible by the development of a network of operators trained to collect wolf DNA samples, with the intent to repeat the sampling over time. However, the costs of this sampling scheme make it impossible to repeat every year and thus follow the development of the population over time. In this sampling scheme, field activities are mainly managed by institutional personnel, who in turn are paid by their respective institutions. Therefore, the main expenses incurred directly by the monitoring program are the laboratory costs for genetic analyses.

Here, we investigated how to optimise the wolf monitoring program in the Italian Alps both from both search effort and DNA analysis perspectives by subsampling the data collected during 2020–2021 (Marucco et al., 2023). Our work aimed to identify sampling strategies that reduce the effort associated with wolf surveys in the Italian Alps without jeopardising the reliability of the SCR estimates. We quantified the consequences of subsampling on the accuracy and precision of the population size estimate in two scenarios: (i) a reduction of the number of NGS successfully analysed in the genetic laboratory, with no reduction in search effort; (ii) a reduction in search effort, both in terms of the number of transect searched and the number of visits per transect, with a corresponding reduction in the number of samples analysed. The first scenario only decreases laboratory costs, while the second scenario decreases costs at all stages, from data collection to DNA analysis. We expected the precision of the population size estimates to decrease with increasing levels of subsampling in all scenarios. Data sparsity can lead to bias in SCR estimates (Efford et al., 2016), and we expected this to manifest in the most extreme subsampling scenarios, where a substantial proportion of samples was lost to the analysis. Our goal with the above line of inquiry was to inform planning and facilitate long‐term monitoring for population size estimation for wolves in the Italian Alps and elsewhere.

## 2 MATERIALS AND METHODS

### 2.1 Sampling design

**FIGURE 1:**
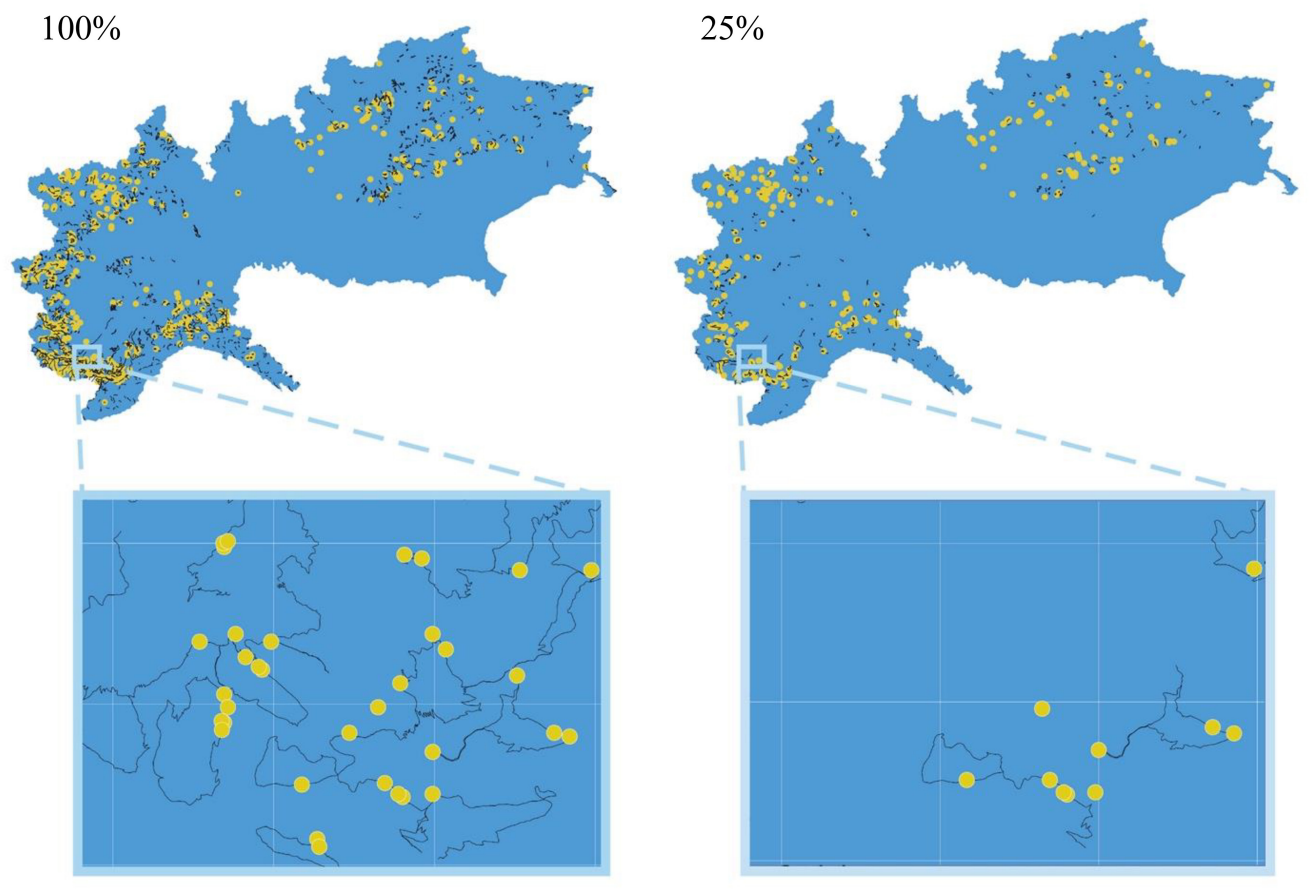
Example of subsampling of the wolf data collected from October 2020 to April 2021 throughout the Italian Alpine regions (blue background). On the left is the full dataset, with 100% of the search transects (black lines) and all genotyped samples (yellow dots). On the right is a subsampled dataset, where 25% of the search transects and associated genotyped samples were retained, the white grid‐lines indicate the 5 × 5 km detector grid used in the analysis. Lower panels present a zoom in of a specific area to better visualise the consequences of subsampling. Note that opportunistic samples (not associated with a search transects) are kept when subsampling, as visible from the isolated yellow dot in the lower right panel.

Our subsampling study used the dataset from the 2020/21 Italian Alpine wolf survey (Marucco et al., 2023). The sampling period started on 1 October 2020 and ended on 30 April 2021 and was mostly based on systematic transects (Figure 1a) along roads and trails used by wolves. We used linear transects to detect and collect scats, aided by the detection of snow tracks of wolf packs. Tracks were used to maximise the probability of identifying all members of a pack and develop a pedigree of the overall population (Marucco et al., 2009, 2012).

The study area was divided into 10 × 10 km grid cells, classified into two categories: cells with pre‐existing information on the presence of wolf packs were intensively sampled with search transects that were repeated at least six times (once a month), while transects in neighbouring cells or areas of the new expansion were repeated at least three times (once every 2 months). The variation in the number of transects and the different number of repetitions resulted in large differences in search effort, with distance searched ranging from 0 to 658 km (mean = 10 km) per cell, and the number of repetitions per transects ranging from 1 to 29 (mean = 4.95) (Figure S5). In addition to this systematic sampling, opportunistic sampling (unplanned searches) was carried out everywhere within the study area, including in areas without confirmed wolf presence. Opportunistic sampling was carried out by the same trained operators involved in the systematic collection, but it could not be quantified due to its opportunistic nature.

Pack pedigrees were constructed from the successfully genotyped NGS as described in Marucco et al. (2023). This allowed us to categorise detected individuals' social status: ‘reproductive individual’ (RI, dominant individuals who reproduce inside the pack), ‘offspring’ (members of a pack with shared genetic heritage with RI), and ‘other’ (members of a pack with no genetic relationship or dispersing individuals).

### 2.2 Spatial capture‐recapture model

Spatial Capture‐Recapture models are hierarchical models that allow for the estimation of population size and density (Borchers & Efford, 2008; Royle & Young, 2008). For this study, we used the same Bayesian SCR model as Marucco et al. (2023). SCR models rely on multiple recaptures of the same individuals at different locations in space to estimate the distribution of the individuals' activity centre (AC) across the habitat. Because the size of the total population is unknown, both the number of individuals and their ACs are treated as latent variables. To estimate AC locations, including those of undetected individuals, SCR models are composed of two sub‐models, an observation model and a density model.

(i) The observation model describes the probability of detecting an individual i at detector *j* as a function of the individual's position relative to the detector. This probability is commonly modelled as a half‐normal function of the distance between the individual's AC and the detector:

$$p_{\mathrm{ij}}=p_{0}\exp\;\left(\frac{-d_{\mathrm{ij}}^{2}}{2\sigma^{2}}\right)$$

with *p*_0_ the baseline detection probability, i.e., the probability to detect the individual if its AC coincides with the detector's position, and the scale parameter *σ* usually considered a measure of home range size during the sampling period (Royle et al., 2014). We used a 5 km‐resolution grid covering the entire Italian Alpine region and used grid centroids as detectors. To account for factors affecting the probability of collecting a wolf DNA sample, we used multiple covariates to model detectors and individual‐specific baseline detection probabilities. We used cumulative snowfall (snow), human population density (humpop), transect searcher experience (searcherexp) and transect length (transect_L): see Marucco et al. (2023) for details:

$${p_{0}}_{\mathrm{ij}}={p_{0}}_{{\mathrm{sex}}_{i}{\text{status}}_{i}}+\beta {\text{snow}}_{j}+\beta {\text{humpop}}_{j}+\beta {\text{searcherexp}}_{j}+\beta \text{transect}\_L_{j}$$

The encounter frequency (*y*_*ij*_) of individual *i* at detector *j* was modelled as a binomial process, following Milleret et al. (2018), where each detector grid cell was divided into 25 sub‐cells of 1 × 1 km:

$$y_{\mathrm{ij}}\sim \text{Binomial}\left(p_{\mathrm{ij}}z_{i},{\text{size}}_{j}\right)$$

and size_*j*_ refers to the number of sub‐cells associated with detector *j*. This formulation means that all detections were aggregated to the closest sub‐cells. See Marucco et al. (2023) for more details.

(ii) The density model describes how individual ACs are distributed across the available habitat *S*. To account for individual ACs that might be situated outside of the sampled area, we considered a 30 km buffer around the sampled region defined by the detector grid to define *S*. Density was then modelled across *S* as an inhomogeneous Binomial Point Process (Zhang et al., 2023) with intensity:

$$I_{h}=e^{\beta_{\text{wolf}\_\text{pres}}*\text{wolf}\_{\text{presence}}_{h}+\beta_{\mathrm{hum}\_\mathrm{pop}}*\mathrm{hum}\_{\mathrm{pop}}_{h}+\beta_{\text{forest}}*{\text{forest}}_{h}+\beta_{\mathrm{br}}*\text{bare}\_{\text{rock}}_{h}+\beta_{\text{herb}}*{\text{herbaceous}}_{h}}$$

where *I*_*h*_, the point‐process intensity in habitat grid cell *h*, is a log‐linear function of the historical presence of wolves (wolf_presence), human population density (hum_pop), the percentage of forest (forest), bare rock (bare_rock), and herbaceous cover (herbaceous).

Following the data augmentation approach (Royle et al., 2013), undetected individuals are added to the population of detected individuals. Individual state is then modelled using a Bernoulli state variable *Z*, which takes value 1 if the individual is part of the population and 0 otherwise:

$$z_{i}\sim \text{Bernoulli}\left(\psi \right)$$

where *ψ* is the probability for an individual from the augmented pool of individuals belong to the population. Population size (*N*) is then obtained by summing over the vector *Z*:

$$N=\sum_{i=1}^{M}z_{i}$$

Using the information provided by the population pedigree, we were also able to model individual sex and social status as:

$${\mathrm{sex}}_{i}\sim \text{Bernoulli}\left(\rho \right)$$

$${\text{status}}_{i}\sim \text{Categorical}\left(\theta_{\mathrm{sex}}\right)$$

where *ρ* is the proportion of males in the population, and *θ*_sex_ is a sex‐specific vector representing the proportion of individuals in each social status category ($\Sigma \theta_{\mathrm{sex}}=1$).

### 2.3 Data subsampling

To optimise the wolf monitoring in the Italian Alps, we considered two subsampling scenarios.

#### 2.3.1 NGS subsampling

This simulation scenario aimed to quantify the consequences of randomly reducing the number of samples genotyped to reduce laboratory costs. To do so, we randomly subsampled the dataset, retaining 25%, 50%, or 75% of the genotyped samples.

#### 2.3.2 Search effort subsampling

To investigate the effect of reducing search effort and thus reduce overall costs, from sample collection to laboratory costs, we artificially subsampled recorded search transects and associated samples along one or both of two dimensions: (i) *transect subsampling*, a reduction in the number of transects and altering the spatial coverage of the survey, and (ii) *repetition subsampling*, a reduction in the number of repeated visits of each transect retained, thus reducing the intensity of the search effort. Practically, this subsampling procedure followed three steps. First, given the uncertainty regarding whether a sample was obtained systematically or opportunistically, and whether the associated effort was documented or not, a classification method was devised. Samples positioned within 500 meters of each transect were designated as systematic, while those positioned beyond this distance were classified as opportunistic. Subsequent subsampling was exclusively conducted on systematic samples (Milleret et al., 2020). Second, to subsample transects, we randomly kept 25%, 50%, 75%, or 100% of the overall 1179 transects. Transect subsampling was random but applied independently in each provincial administrative unit to provide comparable guidelines for institutions coordinating surveys locally. Finally, we randomly retained 3, 6, or all repetitions for the retained transects. We filtered out systematic samples that did not match both the date and location of the retained search transects after each subsampling. Note that following this subsampling procedure, all repetitions were retained for transects with less than 3 or 6 repetitions, depending on the scenario explored. Also note that all opportunistic samples were retained in all search effort subsampling scenarios.

### 2.4 Model fitting and evaluation

Together, the NGS subsampling scenarios (25%, 50%, or 75% of genotyped samples retained) and the search effort subsampling scenarios (25%, 50%, 75%, or 100% of the transects and 3, 6, or all repetitions retained) resulted in 14 different subsampling scenarios. We repeated the random subsampling process a hundred times for each scenario to capture variability among replicates. This led to a total of 1400 SCR datasets. We fitted the SCR model presented above to each subsampled dataset using nimble version 0.10.2 (de Valpine et al., 2017) and nimbleSCR (Bischof et al., 2022) in R version 4.1.3 (R Development Core Team, 2022). We ran 4 chains of 20,000 iterations each, including a 10,000 burn‐in, resulting in a total of 40,000 posterior MCMC samples per model. We assessed model convergence using the Gelman‐Rubin diagnostic (*R̂* < 1.1, Gelman & Rubin, 1992) and by visually inspecting trace plots from randomly selected models.

We evaluated the performance of the SCR model in each scenario based on estimates of population size *N*. We calculated the relative difference (RD) with the SCR model fitted to the full dataset (Marucco et al., 2023):

$$\mathrm{RD}=\frac{\bar{N}-N}{N}$$

where $\bar{N}$ is the posterior mean estimate of the considered simulation replicate, and *N* is the posterior mean estimate from the full dataset. We used the relative difference as a measure of accuracy instead of relative bias since the true value of the parameter is unknown. As a measure of precision, we used the coefficient of variation (CV):

$$\mathrm{CV}=\frac{\mathrm{sd}\left(N\right)}{\bar{N}}$$

where sd(*N*) is the standard deviation of the posterior distribution, and *σ* is the posterior mean estimate of the considered simulation replicate.

## 3 RESULTS

### 3.1 Model convergence

In all scenarios, the proportion of models converged after 20,000 iterations decreased as subsampling increased. We observed the lowest number of models converged for the scenario retaining 25% of all genotyped samples (Table 1). We observed the same pattern in the search effort subsampling scenario, although the proportion of models converged was much higher, even in the scenario with the highest level of subsampling (25% of transects retained and max 3 repetitions, Table 1). Models that did not converge were removed from further analysis and comparisons.

**TABLE 1:** Number of SCR models (out of 100 replicate simulations) that converged for each subsampling scenario (in rows) and for each percentage of samples or transects retained (in columns). NGS subsampling consisted in a random reduction of the proportion of genotyped samples available. For the transects subsampling, we randomly retained 25%, 50%, 75%, or 100% of the transects and 3, 6, or all the repeated visits for the transects retained and analysed the associated samples. We also considered scenarios where we performed an additional removal of transects to retain a maximum number of 3 or 6 transect repetitions.

Scenarios	Maximum number of repetitions retained	% Samples or transects retained			
		25%	50%	75%	100%
NGS subsampling	–	35	88	96	–
Transects subsampling	3	85	88	96	96
	6	85	97	98	99
	All	97	99	100	–

### 3.2 NGS subsampling

**FIGURE 2:**
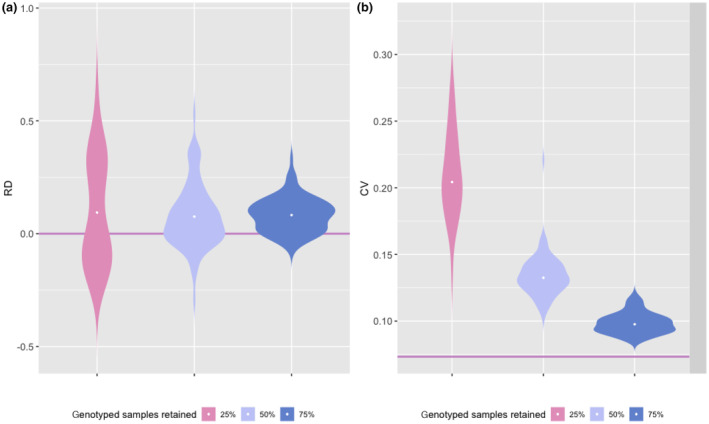
(a) Relative difference (RD) and (b) coefficient of variation (CV) of wolf abundance estimates (*N*) for NGS subsampling (retaining 25%, 50%, and 75% of the full dataset) over 100 replicated datasets, the ones that converged are represented here as a violin plot with the median as a white dot. The pink line represents values obtained when analysing the full dataset model (100% NGS), i.e., a relative difference of 0 and a CV of 0.07.

Retaining 25%, 50%, or 75% of the systematically collected genotyped samples and all opportunistic samples reduced the mean number of individuals detected by 64.8%, 38.3%, and 7.5%, respectively (Tables 2 and S2). Similarly, the mean number of detections per individual decreased, especially when only 25% of samples were retained. The maximum number of detections per individual was also strongly affected. In the 25% scenario, the value fell to 2.9, compared to 5 in the full dataset (Table 2). This resulted, as expected, in a large variation (RDrange = −0.35–0.63) in the population size estimate among replicated SCR datasets, although the mean relative difference was still close (RD = 0.13) to the estimate of the full dataset model (Figure 2a). Overall, SCR models fitted to subsampled genotyped NGS produced slightly higher (mean RD = 0.1, Figure 2a) and less precise population size estimates (Figure 2b) compared to the model fitted to the full dataset. When retaining only 25% of the samples genotyped, the CV reached approximately 20, compared to less than 5% for the full dataset (Figure 2b).

**TABLE 2:** Summary of subsampled datasets (mean ± sd) after retaining 25%, 50%, 75%, and 100% of the genotyped NGS. NDet: Number of successfully genotyped samples, AveRec: Mean number of detections per individual detected, MaxDet: Maximum number of detections per individual, IDs: Total number of individuals; Females: Number of females; Males: Number of males; RI: Number of reproductive individuals; Offspring: Number of individuals sharing part of their DNA with the RI of the same pack; Other: Number of individuals of a pack with no relatedness with RI. Note that not all detected and genotyped individuals could be assigned to one of the sex or social status categories considered.

NGS	25%	50%	75%	100%
NDet	173.76 (±3.48)	327.69 (±5.1)	465.89 (±5.57)	593
AveRec	1.09 (±1.24)	1.18 (±1.08)	1.25 (±1.07)	1.32
MaxDet	2.92 (±0.56)	3.76 (±0.6)	4.49 (±0.52)	5
IDs	158.18 (±4.34)	277.01 (±5.54)	370.52 (±5.99)	449
Females	79.75 (±5.26)	138.09 (±5.49)	183.79 (±5.99)	222
Males	75.15 (±5.66)	132.03 (±5.49)	176.47 (±4.95)	213
RI	45.08 (±5.02)	75.73 (±4.27)	95.96 (±3.56)	111
Offspring	51.12 (±4.61)	89.28 (±5.37)	121.86 (±5.05)	149
Other	21.54 (±3.36)	38.61 (±3.83)	52.32 (±3.09)	63

### 3.3 Search effort subsampling

#### 3.3.1 Transects subsampling

Retaining 25%, 50%, or 75% of the transects reduced the total number of samples by 48%, 31%, and 15% and the number of individuals detected by 42%, 27%, and 13%, respectively (Tables 3 and S1). Similar trends were found for both males and females, and wolves of different social status. The maximum number of detections remained above 4 in all scenarios. The numbers of detections and individuals detected by sex and social status in the 75% scenario showed values close to the full dataset (Table 3).

**TABLE 3:** Summary of subsampled datasets (mean ± sd) after retaining 25%, 50%, 75%, and 100% of the search transects and 3, 6, and all repetitions of the searches. Effort: Km searched; NDet: Number of NGS; AveRec: Mean number of detections per individual; MaxDet: Maximum number of detections per individual; IDs: Total number of individuals; Females: Number of female individuals; Males: Number of male individuals; RI: Number of reproductive individuals; Offspring: Number of individuals sharing DNA features with the RI of the same pack; Other: Number of individuals of a pack that share no relatedness with RI. Note that not all detected and genotyped individuals could be assigned to one of the sex or social status categories considered.

Spatial coverage	25%			50%			75%			100%		
Repetitions	3	6	All	3	6	All	3	6	All	3	6	All
Effort	5159 (±178)	8570 (±323)	11,553 (±639)	10,507 (±188)	17,441 (±407)	22,143 (±681)	15,741 (±191)	26,178 (±371)	31,294 (±551)	21,097 (±42)	35,077 (±47)	40,725
NDet	261.2 (±6.8)	286.1 (±8.8)	310.1 (±10.9)	302.9 (±8.9)	350.8 (±9.8)	406.9 (±11.5)	342.8 (±8.4)	408.7 (±8.5)	502.5 (±11)	383.8 (±7.9)	464.9 (±4.1)	593
AveRec	1.2 (±0.6)	1.2 (±0.8)	1.20 (±0.8)	1.2 (±0.8)	1.2 (±0.7)	1.2 (±0.7)	1.2 (±0.8)	1.2 (±0.7)	1.3 (±0.7)	1.2 (±0.8)	1.2 (±0.7)	1.32
MaxDet	4.2 (±0.4)	4.2 (±0.4)	4.2 (±0.4)	4.4 (±0.5)	4.5 (±0.5)	4.6 (±0.5)	4.6 (±0.5)	4.7 (±0.42)	4.9 (±0.3)	4.7 (±0.4)	5 (±0)	5
IDs	222.8 (±5.9)	241.9 (±6.9)	258.2 (±8.5)	255.2 (±7.4)	289.4 (±7.2)	328.9 (±8.7)	285.4 (±6.7)	330.7 (±6.4)	392.4 (±5.1)	314.7 (±6.2)	369.1 (±2.9)	449
Females	111.4 (±4)	120.8 (±4.5)	128.9 (±5.3)	128.2 (±4.8)	143.9 (±4.7)	164.1 (±5.6)	143.5 (±4.3)	163.9 (±4.4)	194.8 (±5.1)	157.6 (±3.7)	182.4 (±2)	222
Males	106.2 (±3.6)	115.5 (±4.2)	124 (±4.7)	120.5 (±4.4)	136.9 (±4.3)	156.6 (±5.1)	133.9 (±4.1)	155.9 (±3.4)	186.5 (±4.8)	147.7 (±4)	173.4 (±2.3)	213
RI	53.6 (±3.2)	59.2 (±3.6)	64.8 (±4)	63.4 (±3.7)	72.5 (±3.9)	83.0 (±3.7)	72.1 (±3)	83.2 (±2.7)	98.74 (±3.2)	80 (±2.9)	92.2 (±1.3)	111
Offspring	64.3 (±3.3)	70.7 (±4.2)	79.6 (±4.3)	74.9 (±4.2)	86.9 (±4.2)	104.6 (±5.5)	85.1 (±4.3)	101 (±3.5)	128.9 (±4.5)	95.7 (±3.5)	114.3 (±1.5)	149
Other	31.9 (±2.5)	34.4 (±2.9)	34.6 (±2.8)	37.8 (±2.7)	42.3 (±2.7)	45.8 (±2.8)	42.9 (±2.6)	49.36 (±2.6)	54.7 (±2.5)	47.8 (±2.5)	56.5 (±1.5)	63

**FIGURE 3:**
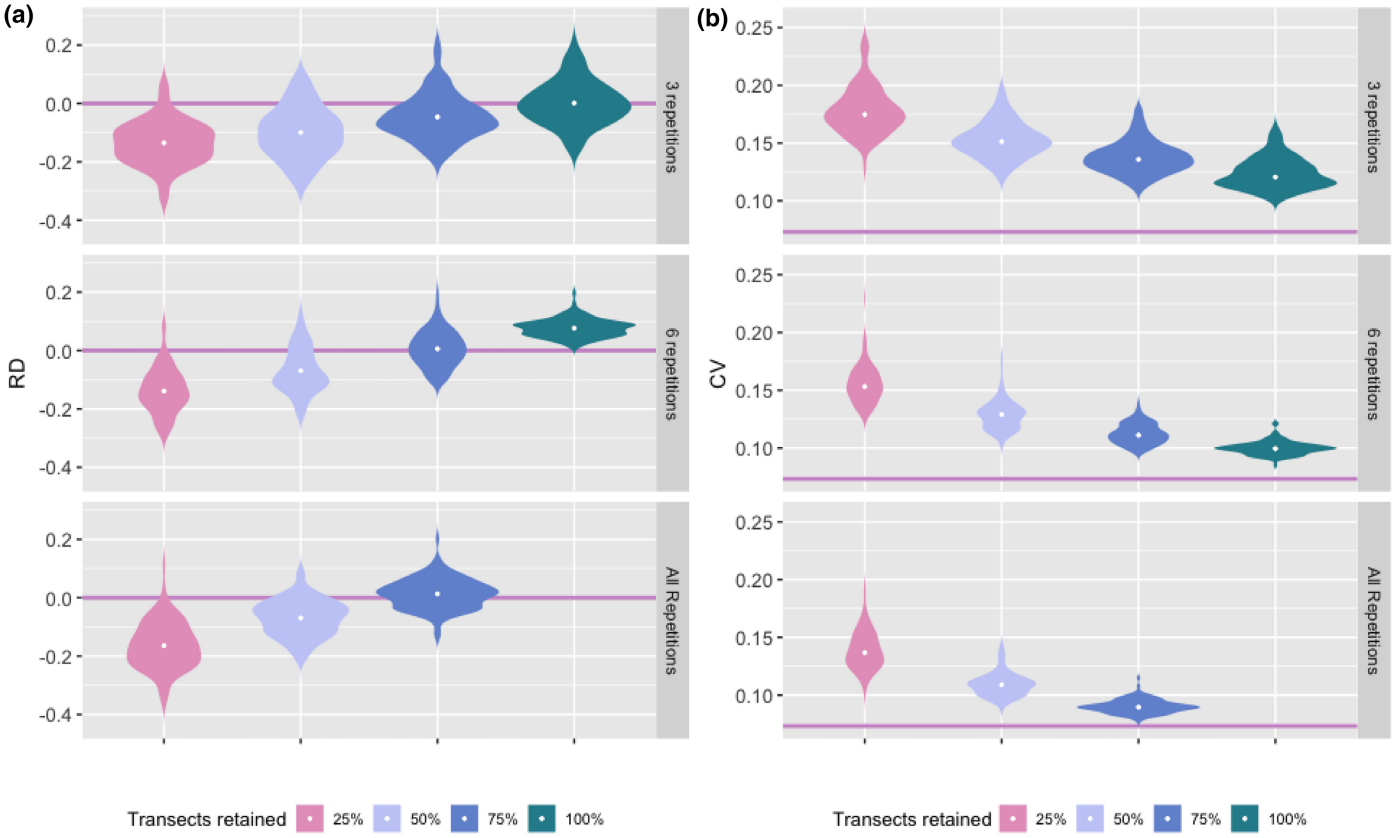
(a) Relative difference (RD) and (b) coefficient of variation (CV) of wolf abundance estimates (*N*), for different proportions of search transects retained (25%, 50%, 75% and 100% of transects) and repetitions per search transect (3, 6 or all repetitions). Violin plots represent the distribution of the relative difference over converged subsampling iterations, with the median value as a white dot. Pink lines represent the estimate from the model fitted to the full dataset.

Subsampling transects led to a decrease in the mean posterior population size estimate (Figure 3a). The mean relative difference changed from 1.3% when discarding 25% of the transects to −6.9% and −13.5% when discarding 50% and 75% of the transects, respectively. The latter corresponded to a population size estimate that was 250 individuals lower than the approximately 1000 individuals estimated with the full dataset (Figure 3a). As in the NGS subsampling scenario, the precision of population size estimates decreased when subsampling search transects (25% CV = 0.139; 50% CV = 0.109; 75% = 0.09, Figure 3b).

#### 3.3.2 Repetitions subsampling

As expected, the total number of detections and the number of individuals detected were negatively affected when reducing the number of repetitions per transect (Table 3, Figure S4). This decrease was most prominent in the scenario with 100% of the transects retained, where limiting the number of repetitions per transects to 6 or 3 generated on average 20% and 35% fewer detections, and 18% and 30% fewer individuals detected, respectively. In the scenarios discarding the 75% of the transects, differences were less marked, with a 7% and 15% reduction in the number of detections, and a 6% and 14% reduction in the number of individuals detected for the scenarios with a maximum of 6 and 3 repetitions, respectively (Table 3).

Overall, reducing the number of repetitions resulted in decreased precision in population estimates across all levels of transects subsampling. However, it maintained a CV close to that of the original dataset model (Figure 3). For example, in the scenario with 50% of the transects retained, limiting the number of repetitions translated into increasing CV values, from an average of 0.11 to 0.13 and 0.15 in the scenarios with all, 6, or 3 repetitions per transect retained, respectively (Figure 3b).

The increase in CV was not accompanied by a noticeable increase in RD in *N*, with average RD values of −0.1 in the same three scenarios with 50% of the transects retained (Figure 3a). Overall, the RD value tended towards zero as the percentage of transects retained increased with no significant variation between the repetition scenarios (Figure 3a).

Surprisingly, retaining a high number of transects and a high number of repetitions resulted in higher estimates of *N*, compared to the model fitted to the full dataset. In fact, for the scenario where all and 75% of the transects are retained, and with 6 or all repetitions, we obtained positive RDs. The most relevant case is that of 100% transects retained and 6 repetitions, for which we observed an average RD of 0.08.

For all scenarios, *N* and *σ* estimates, with relative, RD and CV, by sex and status can be found in Figures S1–S3.

## 4 DISCUSSION

SCR is a common approach for estimating wildlife population density (Tourani, 2022), but SCR models are not immune to the limitations associated with sparse data. To provide reliable estimates for large populations, these methods usually require large datasets and thus major data collection effort (Dupont et al., 2019; Paterson et al., 2019). Marucco et al. (2023) searched more than 40,000 km of transects over approximately 100,000 km^2^ to generate the first wolf population size estimate for the entire Italian Alps. Here, using this first survey of the population as a starting point, we explored the consequences of reduced sampling effort to help increase cost‐efficiency and facilitate long‐term monitoring.

### 4.1 Subsampling trade‐off

The scenarios that offered the best trade‐off between effort reduction and accurate abundance estimation were the scenarios with 75% of the transects retained and 6 repetitions and the scenario with all 100% transects retained and 3 repetitions per transect (Figure 3a). Implementing the first scenario would lead to an approximately 36% decrease in search effort, from 40,725 to 26,178 (±371) km of transects searched. This translates into the removal of up to 295 of the 1179 transects and 31% of the detections. The second scenario would reduce the search effort by approximately 52% (−21,097 ± 42 km), and result in the loss of 35% of the detections. Despite these substantial reductions in search effort and number of samples analysed, overall abundance estimates were comparable with those from the full dataset.

It is worth noting that, the reliability of population size estimates might also be affected by population size itself. For instance, if population size decreases, this will also lead to a decrease in sample size. Coupling a decrease in the wolf population with reduced sampling effort could exacerbate the challenges of critical data situations, where sparse data lead to unreliable or biased estimates (Marques et al., 2011; Sollmann et al., 2012). Sparse data tend to introduce positive bias in population estimates, meaning that population size may be overestimated (Paterson et al., 2019). As a consequence, this decreases the chances to detect a population decline. Nowadays, identifying the decline, rather than the growth of a population, becomes increasingly relevant. This is true for species for which precise conservation efforts are in place, but even more so for the wolf for which massive management plans are in prospect. However, since effort is recorded, SCR models should be able to assess whether a lower sample size is due to a reduction in search effort or a decrease in diminished population size. Nonetheless, decreased sampling leads to higher uncertainty, reducing the ability to identify substantial population changes.

In our study, the reduction in the number of transects and the consequent decrease in the number of samples did not affect the abundance estimates according to social status. Thus, even reduced sampling led to accurate abundance estimates for the different social classes. Numbers of ‘reproductive individuals’ (RIs) and ‘others’ (Figure S1A) are used to produce estimates of the number of mature individuals (Marucco et al., 2023), a critical parameter for IUCN Red List assessments (IUCN, 2022). The number of ‘RIs’ also allows estimation of the number of packs, which is critical for assessing the conservation status of the species. Reducing search effort while still being able to provide these estimates with high accuracy is thus highly valuable.

### 4.2 Subsampling NGS

Randomly subsampling the number of genotyped samples without considering the associated spatial effort led to fewer individuals detected and fewer spatial recaptures per individual. This resulted in slightly higher population size estimates and a significant loss in precision. This is in line with previous works that found data sparsity can cause biased estimates (Milleret et al., 2020; Paterson et al., 2019; Sun et al., 2014). Schmidt et al. (2022) suggested that a low proportion of individuals with multiple spatial recaptures (<0.3%) could be a signal for inflated estimates of population size. This risk should be avoided, especially for endangered, controversial, or exploited species, such as wolves. Furthermore, the precision of estimates tends to increase with the number of individuals detected (Morin et al., 2018; Schmidt et al., 2022). The scenario with only 25% of the genotyped samples retained had the highest CV of all scenarios tested, exceeding the 0.2 threshold, which was suggested as the maximum CV value to efficiently detect changes in a population (Efford & Boulanger, 2019; Skalski et al., 2010). Finally, a large proportion of the models in this scenario did not reach convergence (65%), and we thus caution against such drastic sample size reduction.

In the SCR framework, density is estimated concurrently with *σ* (individual space use) and *p*_0_ (the baseline detection probability). Unaccounted variation in these parameters has been linked to biased abundance estimates (Borchers & Efford, 2008; Efford et al., 2016). For example, negative bias in the scale parameter estimates has been associated with positively biased densities (Efford et al., 2016; Harmsen et al., 2020). Similarly, negatively biased *p*_0_ estimates result in positively biased density estimates (Milleret et al., 2020; Moqanaki et al., 2021; Paterson et al., 2019). Subsampling NGS regardless of their location resulted in a drastic reduction in the number of detections per individual, leading to changes in the estimates of *σ* and *p*_0_. In particular, the lower estimates of *σ* for females of all social statuses (Figure S3.1), which make up half of the Italian Alpine wolf population (Marucco et al., 2023), might be a reason for the higher *N* estimates after subsampling. Data sparsity after subsampling most likely underlies this underestimation of *σ*, as well as the higher and imprecise population size estimates.

### 4.3 Subsampling search effort

In most search effort subsampling scenarios, reducing the number of transects and retaining 3, 6, or all the transect repetitions, resulted in comparable estimates of *N*. Considering their closeness to the reference value and the fact that the CV of the estimates were always below the recommended threshold, reducing the number of transects seems a more appropriate strategy than subsampling NGS alone. In the original sampling design, transects were placed in proximity to each other to account for the movements of packs and their members throughout the occupied territories and to maximise the probability of detecting individuals at multiple detectors. This feature is maintained when reducing the number of transects if a significant number of transects is retained. In fact, explicitly considering space, i.e. search transects, when subsampling produced more stable and higher values in both the average and maximum number of detections per individual compared to the NGS subsampling scenarios (Tables 2 and 3). Furthermore, and although genetic analysis was the highest cost item, reduction in field collection effort (distance walked) leads to more comprehensive cost savings, including savings in both field and laboratory costs.

The observed underestimation in *N* in scenarios with less effort retained, may lie in the nature of SCR models, where the detections of a given individual are expected to be spatially autocorrelated. The effective removal of one or more transects may mean the total loss of an individual and all its detections, but without altering the probability of detection of the other individuals, which in turn may result in a higher estimate of the apparent probability of detection and thus lower population size estimates. However, this potential issue should in theory be mitigated by using the length of the transects as a covariate on the baseline probability of detection, to consider differences in effort between detection grid cells (Milleret et al., 2020), stressing the importance of accounting for heterogeneity in search effort in SCR models (Moqanaki et al., 2021).

Reducing the number of repeated searches along transects is another effective way to reduce monitoring effort. Reducing to 3 repetitions per transect caused no major deviation in the estimates. Interestingly, we observed slightly higher population size estimates in the scenario with 100% of the transects retained and a maximum of 6 repetitions per transect compared to the full dataset, while we obtained lower or comparable population size estimates in all other search effort subsampling scenarios. Our model accounted for individual variation in detectability and home range size (i.e., using the sex and status of the individual), and spatial variation in detectability (i.e., linked with search effort, snow conditions, searchers' experience). However, additional sources of heterogeneity in detectability may have remained unaccounted for by the model (Moqanaki et al., 2021), e.g., variability in the genotyping success of samples. Alternatively, subsampling may have introduced additional sources of heterogeneity that we were not able to account for. Since our study used empirical data; we were not able to identify with certainty the causes of this apparent overestimation.

### 4.4 Sampling design recommendations

The current study relied heavily on the accurate quantification of search effort, through the recording of search paths using GPS. This information also enabled the implementation of various subsampling scenarios. Nevertheless, around 30% of the samples genotyped were collected opportunistically and thus lack information on search effort. Opportunistic sampling is important to document areas of new recolonization where systematic transects have not yet been implemented or only occur at low density. When the goal is to estimate the size of wide‐ranging populations, we thus advocate for a more homogeneous distribution of search transects, with the goal to cover as much as possible of the area of interest, i.e., a reduction of the number of transects in areas where they are already present at high density and an increase in areas where they are still few or absent. From the model's perspective, it is also important to obtain information about the absence of the species and not only rely on opportunistic sampling for which we often struggle to quantify effort (Moqanaki et al., 2021).

Indeed, for monitoring programs relying on NGS, maximising spatial coverage rather than sampling the same transect multiple times can significantly enhance the effectiveness and efficiency of data collection. In this study, we used the number of genotyped samples as a metric, but when planning sample collection, one has to take into account the expected genotyping success (which averages 65% in our case) to obtain the desired number of exploitable samples for the analysis.

Our subsampling approach of previously collected data offers opportunities for optimising cost efficiency and assessing the sensitivity of results to different sampling scenarios. By systematically subsampling existing datasets and comparing the outcomes, researchers can determine the minimum sampling effort required to achieve desired levels of accuracy and precision. This information can then inform the design of future monitoring programs, helping to strike a balance between information gain and resource allocation.

## 5 CONCLUSION

Long‐term monitoring programs are needed for efficient wildlife management and, at the same time, are highly demanding (Lindenmayer et al., 2011, 2022; Lindenmayer & Likens, 2009). Our empirical data‐based modelling approach helped us in optimising wolf monitoring in the Italian Alps by adjusting the sampling effort, both in terms of spatial coverage and intensity. We also found that randomly reducing NGS a posteriori, not considering space, was not a good practice. In addition, integrating additional information (camera traps, Chandler & Clark, 2014; dead recoveries, Dupont et al., 2019) to SCR models are also promising alternatives to optimise the monitoring program of wolves in the Italian Alps and should be explored further.

Adaptive management is a dynamic approach that involves testing predictions against observations, enabling iterative recalibration of management strategies at predetermined decision points as learning occurs (Allen & Garmestani, 2015; Williams, 2011). This learning process facilitates the progression of management actions as uncertainty diminishes over time (Williams, 2011). We believe that the adaptive management approach described here can be advantageously applied to wildlife population monitoring elsewhere.

Wildlife monitoring programs usually operate with limited resources. Resources are particularly limiting, when programs are implemented long‐term and over large spatial scales and involve different administrations with different budgets. To cope with these constraints, we propose sampling protocols that allow flexibility in data collection strategies, sampling frequencies, and spatial coverage. This, in turn, accommodates variations in target species and environmental conditions in different regions, as well as economic conditions.

By applying an adaptive management approach to wildlife monitoring activities, as in the wolf monitoring program in the Italian Alps, researchers can improve the scalability, applicability, and impact of monitoring initiatives in different ecosystems and geographical contexts.

## AUTHOR CONTRIBUTIONS

**M. V. Boiani:** Conceptualization (lead); data curation (supporting); formal analysis (lead); methodology (supporting); software (lead); visualization (lead); writing – original draft (lead). **P. Dupont:** Conceptualization (lead); formal analysis (lead); methodology (lead); supervision (lead); writing – review and editing (lead). **R. Bischof:** Conceptualization (supporting); methodology (supporting); writing – review and editing (supporting). **C. Milleret:** Methodology (equal); writing – review and editing (lead). **O. Friard:** Software (lead). **M. Geary:** Methodology (supporting); supervision (supporting); validation (supporting); writing – review and editing (supporting). **E. Avanzinelli:** Data curation (lead). **A. von Hardenberg:** Methodology (supporting); supervision (supporting); writing – review and editing (supporting). **F. Marucco:** Conceptualization (lead); data curation (supporting); funding acquisition (lead); methodology (lead); project administration (lead); resources (lead); supervision (supporting); writing – review and editing (lead).

## FUNDING INFORMATION

LIFE Wolfalps EU Project (LIFE18 NAT/IT/000972) and Research Council of Norway, grant NFR 286886 (project WildMap).

## CONFLICT OF INTEREST STATEMENT

All authors declare that they have no conflicts of interest to disclose.

## Supporting information


Appendix S1.

## Data Availability

Scripts for the simulations are available on GitHub ‘PierreDupont/AlpineWolf’.
