# Supplementary material for: When enough is enough: Optimising monitoring effort for large‐scale wolf population size estimation in the Italian Alps
Source: Ecol Evol. 2024 Aug 21;14(8):e70204. doi: 10.1002/ece3.70204 (PMC11337114; doi:10.1002/ece3.70204)
Supplement: Supplementary file 1 — Appendix S1. [file ECE3-14-e70204-s001.docx]

**Supplementary Information**

1. **NGS subsampling by sex and status**

**
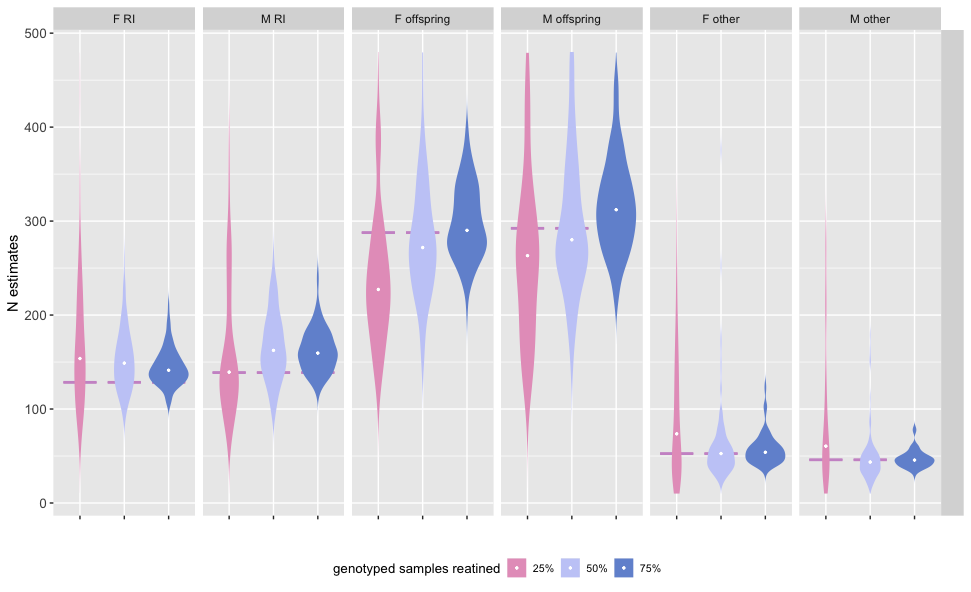
**

**
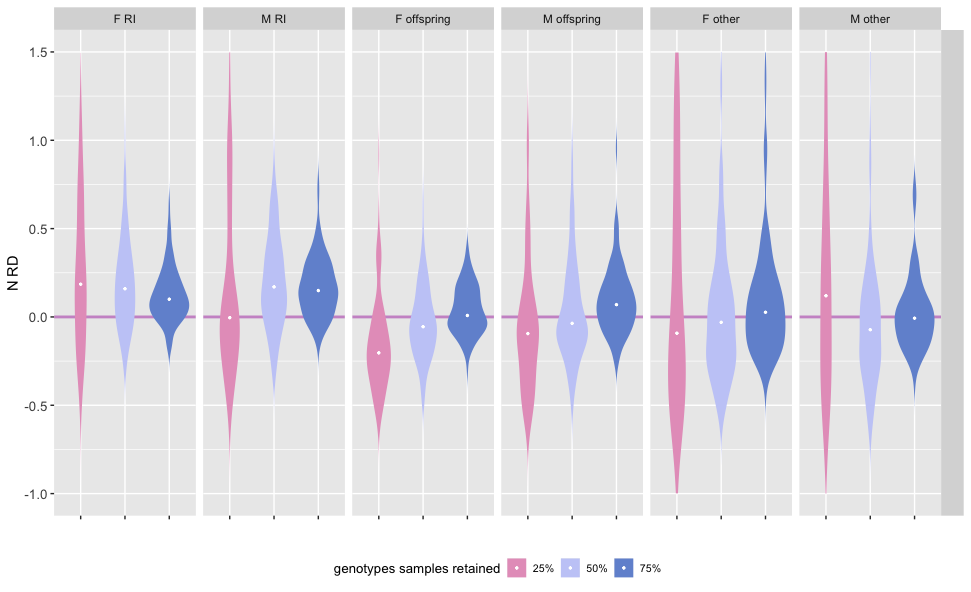
**

**
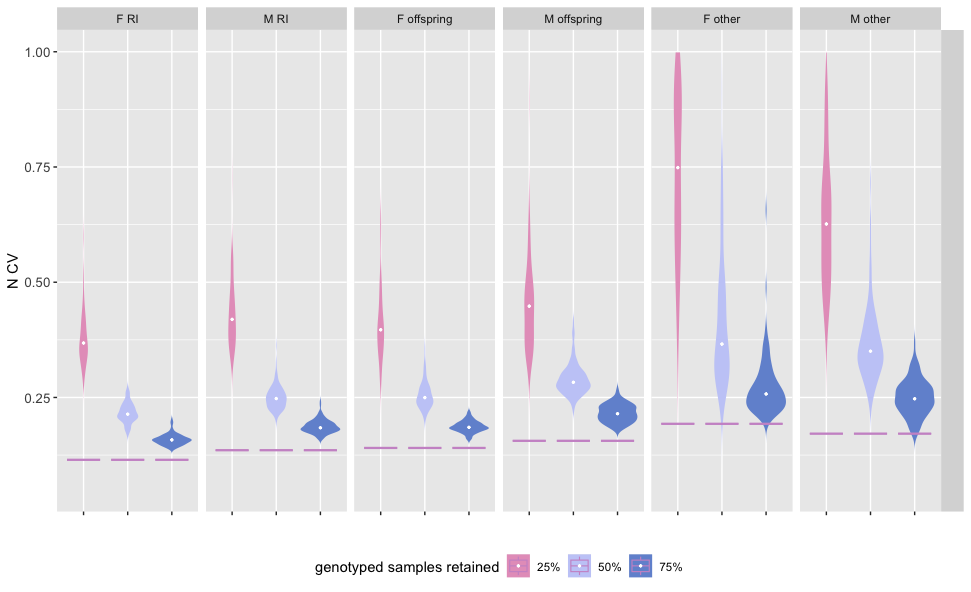
**

Figure 1. A) Population size (*N*), B) Relative difference (RD) and C) coefficient of variation (CV) of wolf abundance estimates (*N*) by sex and social status, for different levels of NGS subsampling (25%,50% and 75%). Violin plots represent the distribution of the parameter over 100 subsampling replicated datasets with the median value as a white dot. Pink lines represent the full dataset model estimate.

1. **Search effort subsampling by sex and status**

**
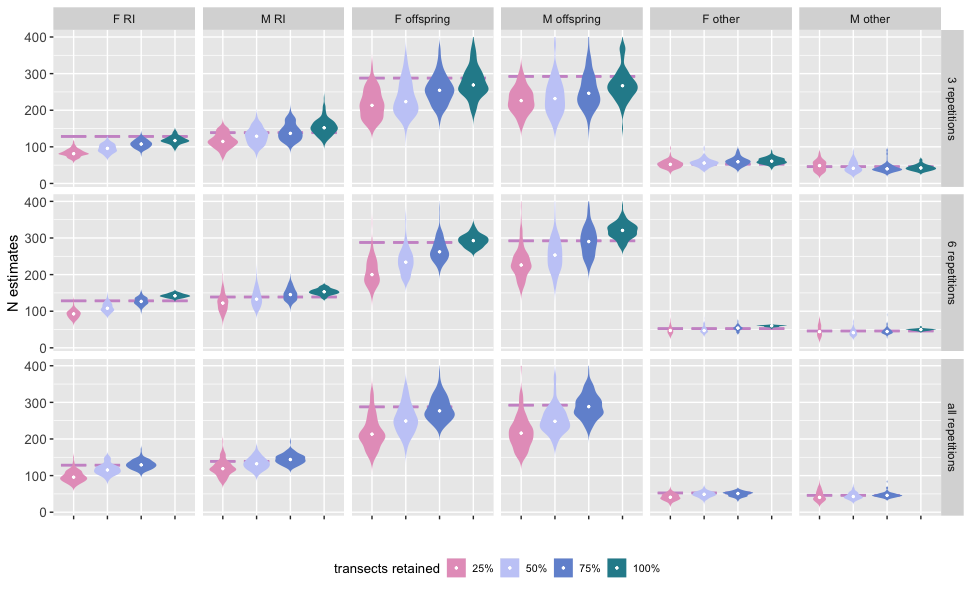
**

**
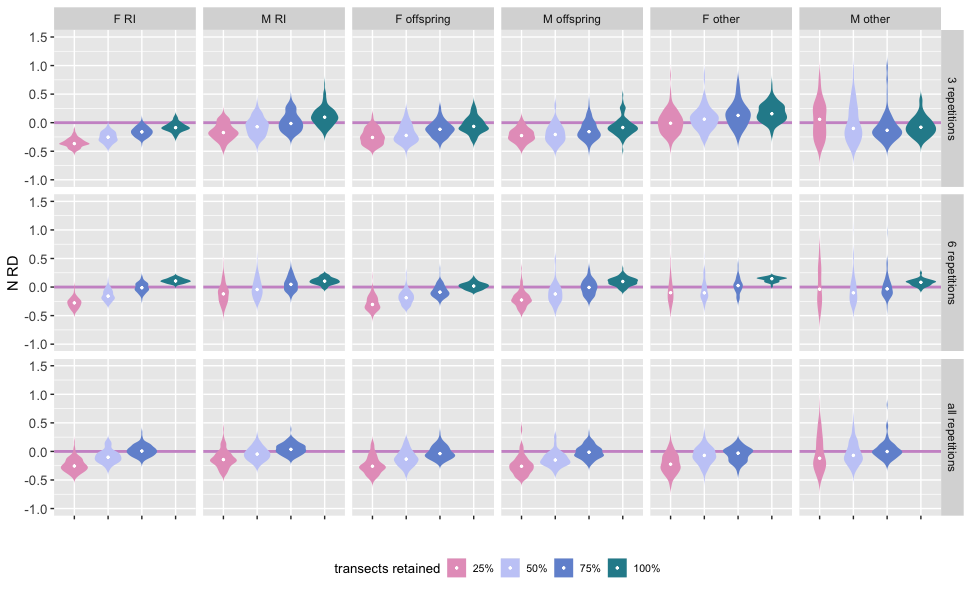
**

**
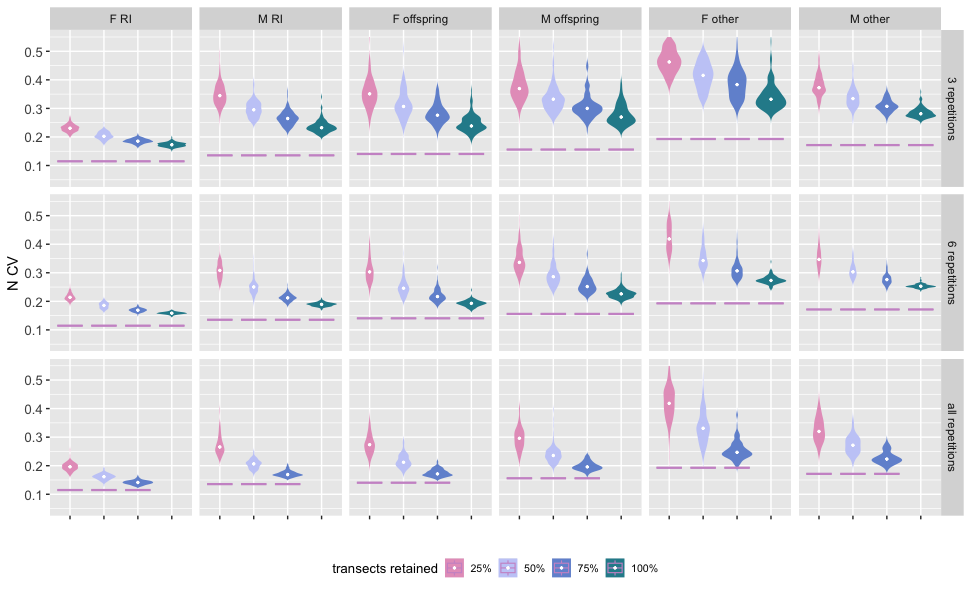
**

Figure 2. A) Population size (*N*), B) relative difference (RD) and C) coefficient of variation (CV) of wolf abundance estimates (*N*) by sex and social status, for different subsampling intensities of the number of search transects (25%,50%, 75% and 100% of transects) and of the number of repetitions per search transect (3, 6 or all transect repetitions). Violin plots represent the distribution of the parameter over 100 subsampling replicated datasets, with the median value as a white dot. Pink lines represent the full dataset model estimate.

1. **Sigma
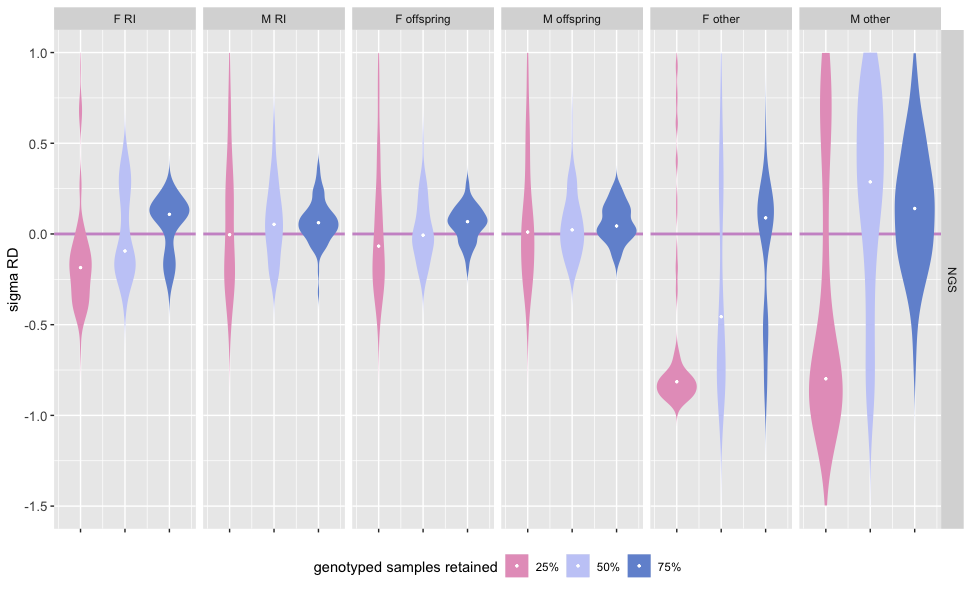
 (**$\boldsymbol{\sigma)}$


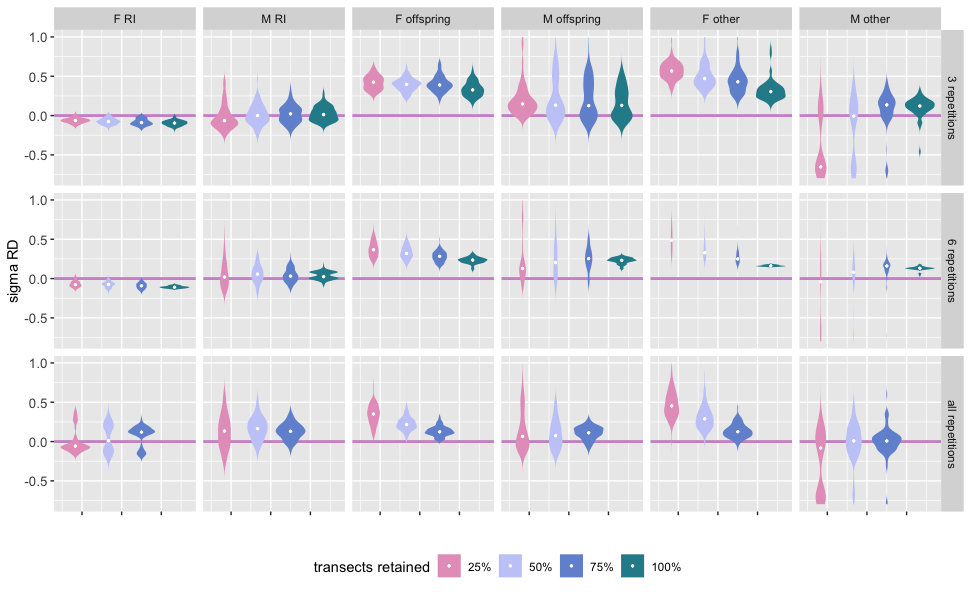


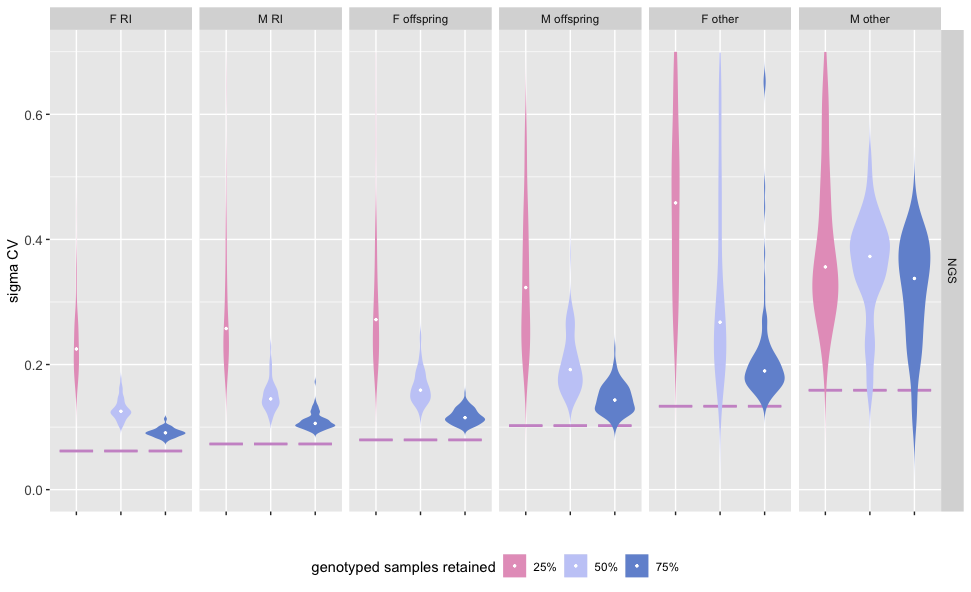

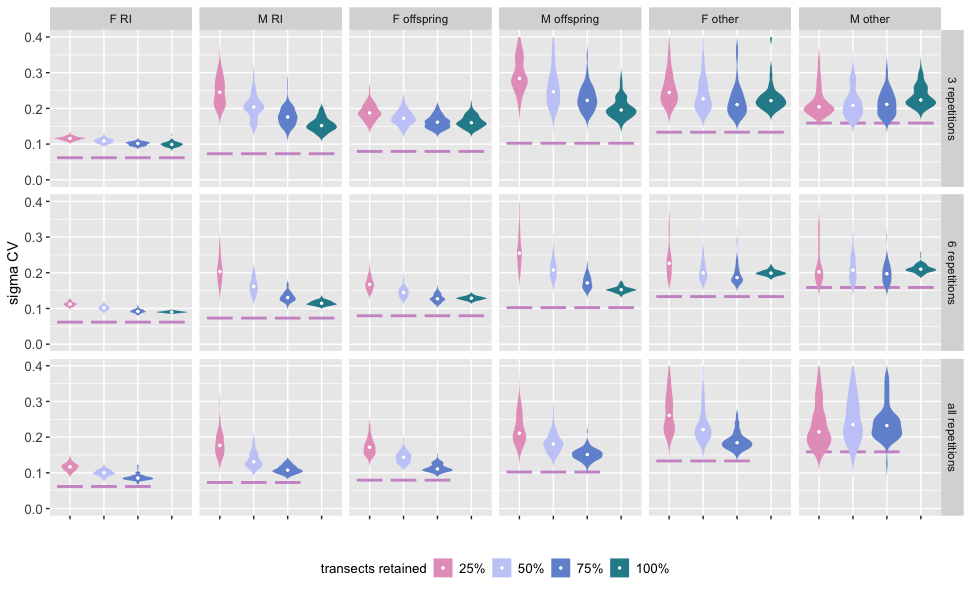


Figure 3. A) Relative difference (RD) of the scale parameter ($\sigma$), for different NGS genotyped subsampling (25%,50%,75%) and for different subsampling intensities B) of spatial coverage (25%,50%, 75% and 100% of transects) and search effort (3, 6 or all transect repetitions), by also considering the effect on sex and status. C) Coefficient of variation (CV) for $\sigma$ represented as above with NGS subsampling and search effort D) subsampling. Violin plots represent the distribution of the posterior mean of the relative difference over 100 subsampling iterations, with the median value as a white dot. Pink lines represent the full dataset model estimate.


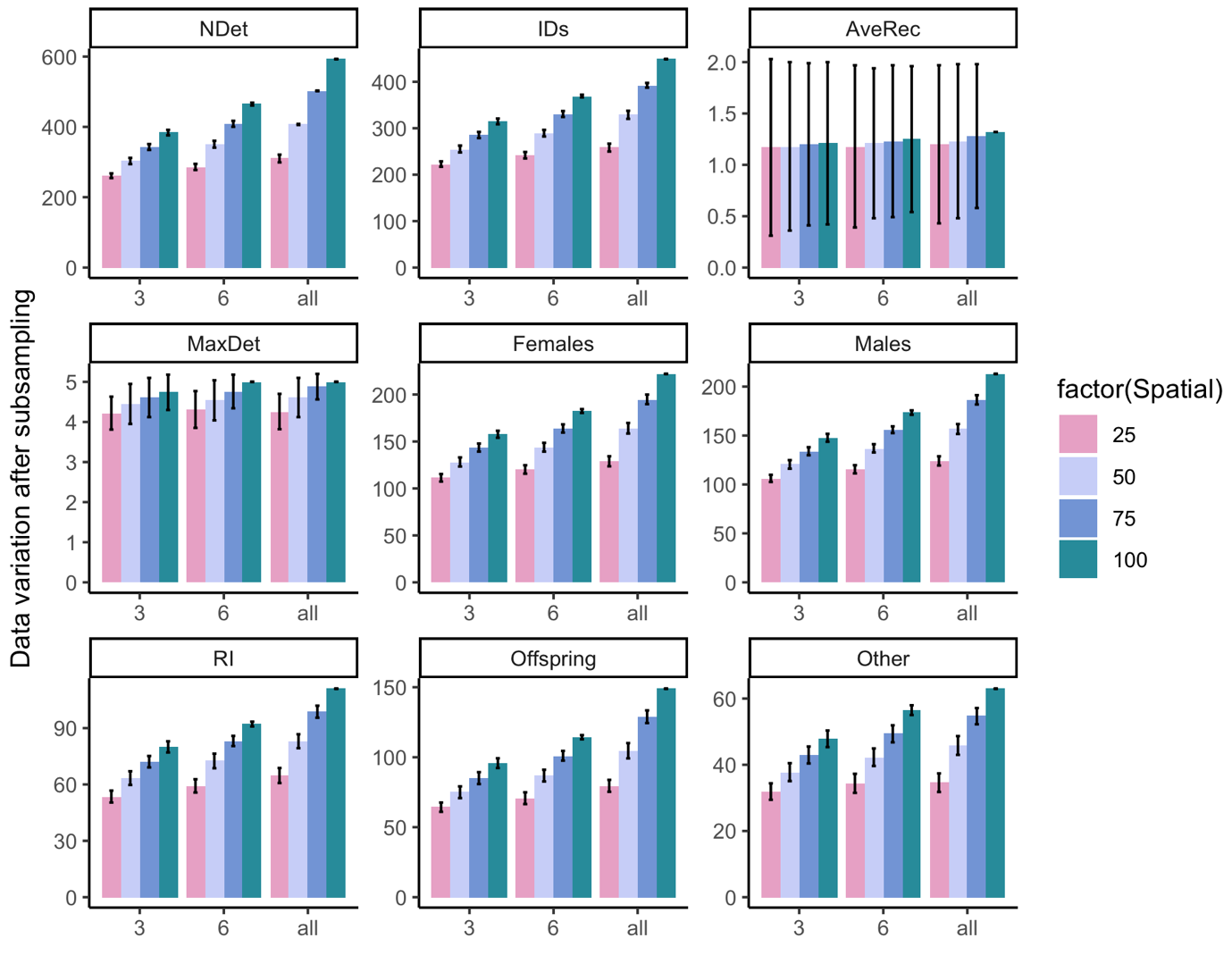


Figure 4. Representation of Table 3 of the main text with bar plot. Summary of subsampled datasets (mean±sd, as error bars) after retaining 25, 50,75 and 100% of the search transects and 3, 6, and all repetitions of the searches, full dataset is 100% transects and all repepetions. Effort: km searched; NDet: number of NGS; AveRec: mean number of detections per individual; MaxDet: maximum number of detections per individual; IDs: total number of individuals; Females: number of female individuals; Males: number of male individuals; RI: number of reproductive individuals; Offspring: number of individuals sharing DNA features with the RI of the same pack; Other: number of individuals of a pack that share no relatedness with RI.

Table 1. Summary of subsampled datasets in % of the data lost from full dataset after retaining 25, 50,75 and 100% of the search transects and 3, 6, and all repetitions of the searches. Effort: km searched; NDet: number of NGS; AveRec: mean number of detections per individual; MaxDet: maximum number of detections per individual; IDs: total number of individuals; Females: number of female individuals; Males: number of male individuals; RI: number of reproductive individuals; Offspring: number of individuals sharing DNA features with the RI of the same pack; Other: number of individuals of a pack that share no relatedness with RI.

| Spatial | 25% | | | 50% | | | 75% | | | 100% | |
| --- | --- | --- | --- | --- | --- | --- | --- | --- | --- | --- | --- |
| Repetitions | 3 | 6 | All repetitions | 3 | 6 | All repetitions | 3 | 6 | All repetitions | 3 | 6 |
| NDet | -55.96 | -51.75 | -47.71 | -48.91 | -40.85 | -31.38 | -42.18 | -31.08 | -15.25 | -35.27 | -21.59 |
| IDs | -50.37 | -46.13 | -42.48 | -43.17 | -35.55 | -26.75 | -36.43 | -26.35 | -12.60 | -29.92 | -17.79 |
| AveRec | -11.36 | -10.61 | -9.09 | -10.61 | -8.33 | -6.82 | -9.09 | -6.82 | -3.03 | -8.33 | -5.30 |
| MaxDet | -15.60 | -13.80 | -14.80 | -11.00 | -9.20 | -7.80 | -7.80 | -4.80 | -2.40 | -5.20 | 0.00 |
| Females | -49.83 | -45.87 | -41.93 | -42.25 | -35.19 | -26.08 | -35.35 | -26.18 | -12.23 | -28.99 | -17.82 |
| Males | -50.13 | -45.78 | -41.76 | -43.44 | -35.70 | -26.45 | -37.11 | -26.77 | -12.44 | -30.66 | -18.57 |
| RI | -51.74 | -46.64 | -41.65 | -42.89 | -34.67 | -25.19 | -35.04 | -25.08 | -11.05 | -27.92 | -16.96 |
| Offspring | -56.82 | -52.56 | -46.59 | -49.67 | -41.69 | -29.77 | -42.89 | -32.18 | -13.46 | -35.73 | -23.29 |
| Other | -49.32 | -45.40 | -45.10 | -40.03 | -32.87 | -27.24 | -31.81 | -21.65 | -13.19 | -24.06 | -10.30 |

Table 2. Summary of subsampled datasets % after retaining 25, 50, 75 and 100% of the genotyped NGS. NDet: number of successfully genotyped samples, AveRec: mean number of detections per individual detected, MaxDet: maximum number of detections per individual, IDs: total number of individuals; Females: number of females; Males: number of males; RI: number of reproductive individuals; Offspring: number of individuals sharing part of their DNA with the RI of the same pack; Other: number of individuals of a pack with no relatedness with RI.

| NGS | 25% | 50% | 75% | Full dataset |
| --- | --- | --- | --- | --- |
| Detections | -70.70 | -44.74 | -21.44 | 593 |
| IDs | -64.77 | -38.31 | -17.48 | 449 |
| AveRec | -17.42 | -10.61 | -5.30 | 1.32 |
| MaxDetID | -41.60 | -24.80 | -10.20 | 5 |
| Females | -64.08 | -37.80 | -17.21 | 222 |
| Males | -64.72 | -38.01 | -17.15 | 213 |
| RI | -59.39 | -31.77 | -13.55 | 111 |
| Offspring | -65.69 | -40.08 | -18.21 | 149 |
| Other | -65.81 | -38.71 | -16.95 | 63 |


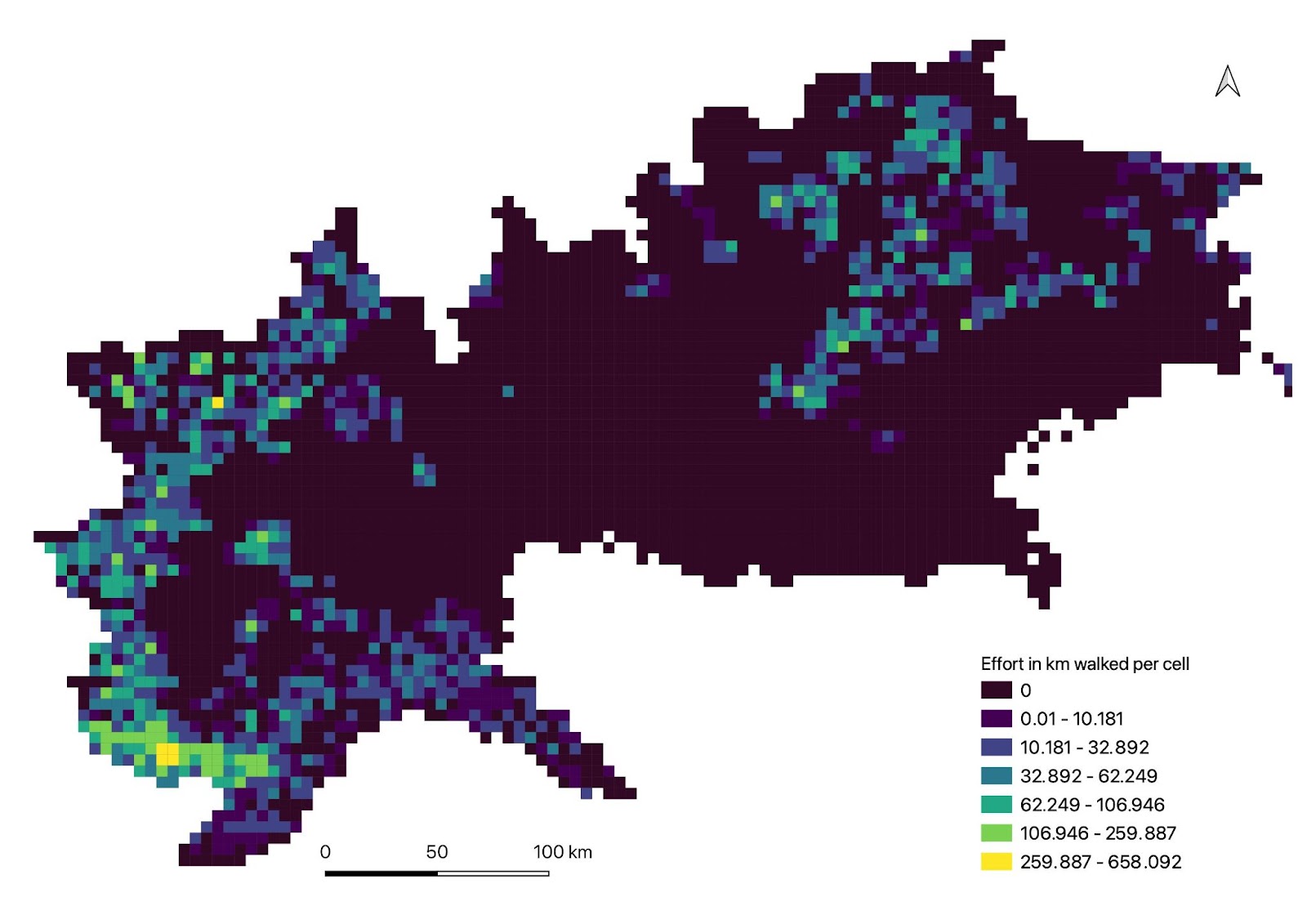


A


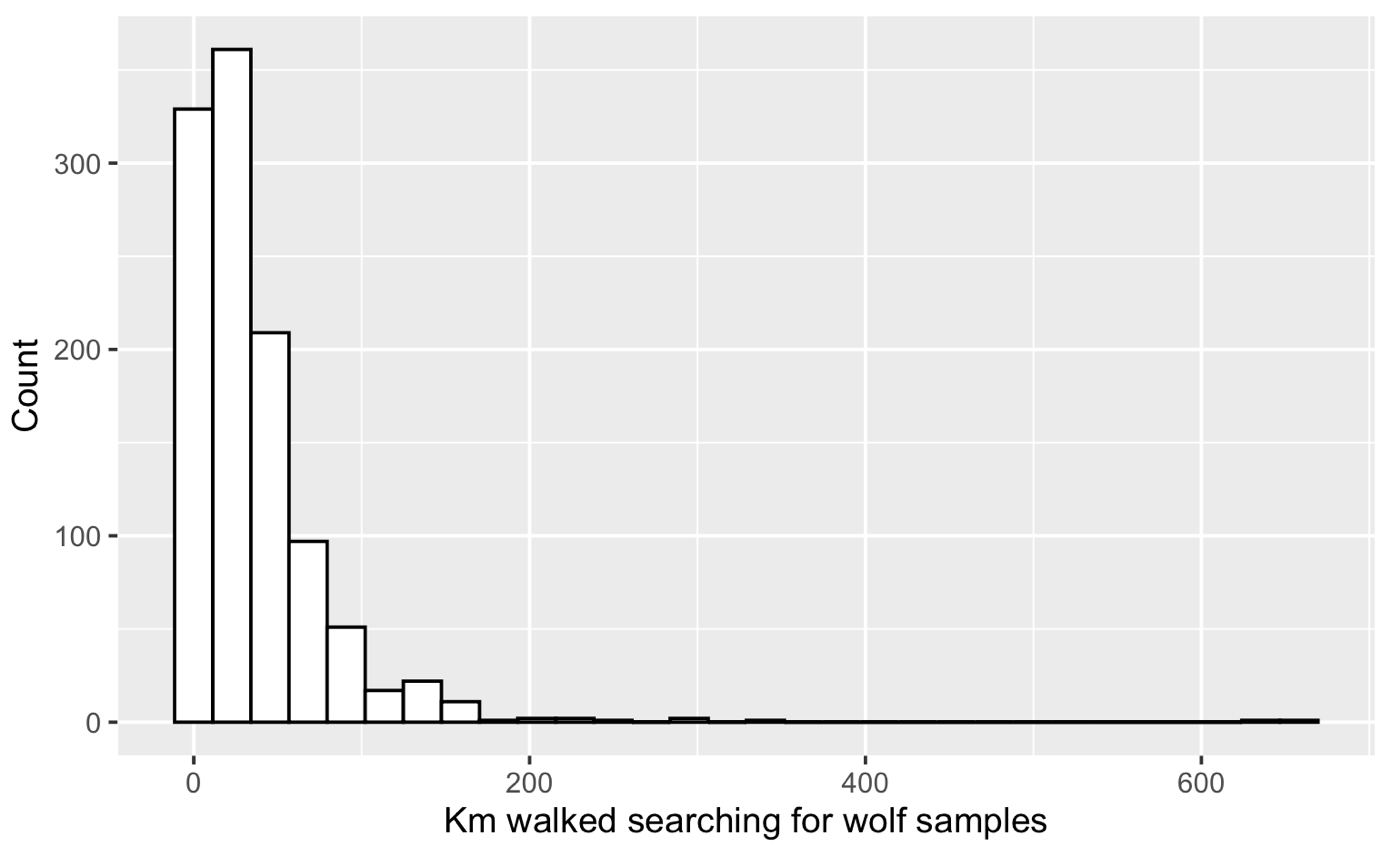


B

Figure 5 A) Map of the Alpine region representing the record length (in kilometres) of the searches to perform NGS during the Italian Alps wolf monitoring survey of 2020-2021. B) Histogram representing the distribution of search effort per cell expressed as the number of kilometres walked.
